# Infectious Diseases and Secondary Antibody Deficiency in Patients from a Mesoregion of São Paulo State, Brazil

**DOI:** 10.3390/tropicalmed9050104

**Published:** 2024-05-06

**Authors:** Luiz Euribel Prestes-Carneiro, Paula Andreia Martins Carrilho, Danielle Francisco Honorato de Barros Torelli, Jose Antonio Nascimento Bressa, Ana Carolina Gomes Parizi, Pedro Henrique Meireles Vieira, Fernanda Miranda Caliani Sa, Mauricio Domingues Ferreira

**Affiliations:** 1 Imunodeficiencies Outpatient Clinic, Regional Hospital of Presidente Prudente, Presidente Prudente 19050-680, Brazil; pedrovieira@unoeste.br; 2 Master’s Program in Health Sciences, Oeste Paulista University, Presidente Prudente 19050-920, Brazil; danielletorelli@unoeste.edu.br; 3 Haematology Service, Santa Casa de Misericordia of Presidente Prudente, Presidente Prudente 19014-030, Brazil; paula.trevisan@medicos.oncoclinicas.com; 4 Outpatient Clinic of Haematology, Nephrology, and Rheumatology, Oeste Paulista University and Regional Hospital of Presidente Prudente, Presidente Prudente 19050-680, Brazil; josebressa@unoeste.br (J.A.N.B.); parizi.patologia@unoeste.br (A.C.G.P.); f254551@dac.unicamp.br (F.M.C.S.); 5 Laboratory of Medical Investigation Unit 56, Hospital das Clınicas da Faculdade de Medicina da Universidade de Sao Paulo, São Paulo 05403-000, Brazil

**Keywords:** secondary antibody deficiency, hypo-γ-globulinemia, infectious diseases

## Abstract

Our aim was to determine the secondary antibody deficiency (SAD) profiles of patients in a mesoregion of São Paulo state, Brazil, focusing on infectious diseases. Demographic characteristics, and clinical and laboratory data were obtained from electronic files; infections were classified as organ-specific and graded as mild, moderate, life-threatening, and fatal. Non-Hodgkin’s lymphoma (NHL) accounted for 30% of patients, nephrotic syndrome (NS) 25%, chronic lymphocyte leukemia 20%, and multiple myeloma 15%. Patients with NS were younger than those in other groups, and hypo-γ-globulinemia was detected in 94.1%, IgG < 400 mg/dL in 60.0%, IgA < 40 mg/dL in 55.0%, and CD19 < 20 cells/mm^3^ in 30.0%. One hundred and one infections were found; 82.1% were classified as mild or moderate, 7.9% as life-threatening, and 3.0% as fatal. Respiratory tract infections were more prevalent (41.5%), and pneumonia accounted for 19.8%. Lower levels of infections were found in patients with NS compared with NHL (*p =* 0.0001). Most patients progressed to hypo-γ-globulinemia and SAD after treatment with immunosuppressants, and mild and moderate infections were predominant. These therapies are increasing in patients with different diseases; therefore, monitoring hypo-γ-globulinemia and infections may help to identify patients at high risk for severe complications, antibiotic prophylaxis or treatment, and immunoglobulin replacement.

## 1. Introduction

Secondary immunodeficiency (SID) is an impairment of the immune system due to extrinsic factors and underlying medical conditions. SID is up to 30 times more common than inborn errors of immunity (IEI) and can occur as a consequence of hematologic malignancies, autoimmune diseases, immunosuppressive therapies, malnutrition, metabolic disorders, chronic infections, and severe trauma. Furthermore, SIDs are becoming increasingly common as new therapies are available [1]. Several mechanisms induce SIDs. In onco-hematology malignancies, SID is induced due to systemic disorders that include aplastic anemia; hematologic malignancies, such as chronic lymphocytic leukemia (CLL), multiple myeloma (MM), Hodgkin’s disease, and non-Hodgkin’s lymphoma (NHL); graft vs. host disease; and sickle cell disease. One of the leading causes of SID in onco-hematology is iatrogenic disorders caused by biological agents such as chemotherapy, immunosuppressants, corticosteroids, monoclonal antibodies, including anti-CD20 agents, and B-cell differentiation and maturation inhibitors, as well as other conditions; and radiation therapy, splenectomy, and bone marrow ablation before transplant [2]. In a large cohort of onco-hematologic diseases, biological agents have become one of the best early therapeutic options. They block inflammatory pathways, which reduces pathologic inflammation through various mechanisms such as cytokine inhibition, monoclonal cell deletion, and co-stimulatory inhibition [3,4]. Consequently, most of these biological agents cause immunosuppression leading to hypo-γ-globulinemia with decreased antibody production and increased risks of infections [1,2,3,4]. In addition to onco-hematologic malignancies, autoimmune diseases are becoming one of the most prevalent causes of SID and secondary antibody deficiency (SAD). B cells play a pivotal role in both cases [1,5].

Germinal center B cells proliferate quickly and, due to their mutagenesis program, can transform normal cells into cancer cells. Thus, reagents that bind to B-cell surface glycoproteins, such as CD20^+^, have been widely used to target B-cell lymphomas by removing cancerous and non-cancerous CD20^+^ cells [4]. Anti-CD20-mediated B-cell depletion has also been well-documented for the treatment of autoimmune diseases such as nephrotic syndrome (NS), systemic lupus erythematosus, rheumatoid arthritis, immune thrombocytopenia (ITP), autoimmune hemolytic anemia, anti-neutrophil cytoplasmic antibody-associated vasculitis, myasthenia gravis, and autoimmune bullous dermatoses [5].

Rituximab (RTX) and bispecific antibody treatments such as teclistamab targeted against B lymphocytes may induce hypo-γ-globulinemia and reduce antibody production and have been widely reported in different diseases such as onco-hematologic malignancies and autoimmune diseases. Low IgG levels after treatment with RTX have been reported, 27% to 50% in children and 3.5% to 40% in adults [6,7]. Several risk factors have been described to induce hypo-γ-globulinemia after treatment with RTX: A low baseline serum immunoglobulin level; the number of RTX treatment cycles (i.e., longer-term RTX treatment); an association with glucocorticoids; mycophenolate mofetil; cyclophosphamide; purine analogs; fludarabine; younger age in children, and older age in adults. The potential risk for increasing infectious disease complications with monoclonal antibodies used in cancer therapy and autoimmune diseases has been a preoccupation of physicians since their introduction in clinical practice [6,7].

Similar to IEI, infectious diseases are considered one of the hallmarks of patients suspected of having or being diagnosed with SID. With the widespread use of biologic immunomodulatory therapies in different areas of medicine, individuals increase their potential risk of developing infectious complications and death. It is imperative to screen for the risk of infections before and during treatment with biologics, especially anti-CD-20 therapies [8,9]. Infections are the leading cause of death in patients diagnosed with CLL, MM, and NHL who develop SID, up to 50% in patients with CLL, and up to 22% and 33% of patients with MM and LNH, respectively [8]. In a recent cohort of patients with NHL treated with RTX, 86.7% developed recurrent infections, including community-acquired pneumonia, chronic sinusitis (85.7%), and gastroenteritis (42.9%) [6]. In a large cohort of 9253 patients with MM, infections were the leading cause of morbidity and mortality, with a seven-fold increased risk of developing a bacterial infection and a ten-fold risk of viral infections [9].

Immunoglobulin replacement therapy (IGRT) for secondary hypo-γ-globulinemia is increasing worldwide [2]. In developing countries in Latin America, not all doctors are prepared to diagnose SIDs and to deal with hypo-γ-globulinemia after treatment with immunosuppressors. Currently, consensus guidelines are not available, and the European guidelines are followed [10]. In Brazil, immunoglobulin replacement therapy is reserved mainly for regional centers. SIDs are underdiagnosed, although more prevalent than IEIs, and doctors have difficulties directing these patients to reference centers [11]. In the western region of São Paulo state, the Regional Hospital of Presidente Prudente (RH) has been the reference center for immunotherapy since 2014 for patients of the Unified Health System (SUS). Here, our aim was to determine the SAD profile of patients in a mesoregion followed in a public reference center and a private specialized clinic of São Paulo state, focusing on infectious diseases.

## 2. Methods

### 2.1. Regional Characteristics

**Figure 1:**
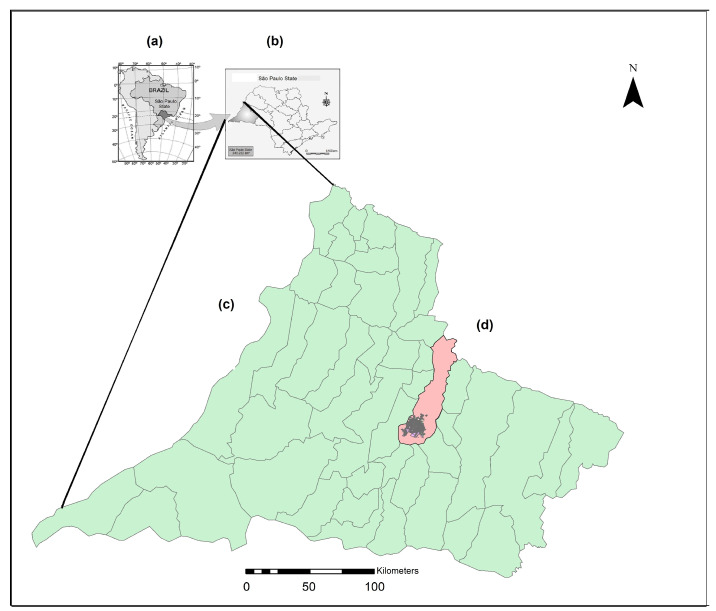
The study setting. (a) country; (b) São Paulo state; (c) Western region of São Paulo state; (d) municipality area of Presidente Prudente.

The western region of Sao Paulo comprises 45 municipalities, and, in 2022, the estimated population was 744,219; it is administered by the Regional Networks for Health Assistance (RNHA11), located in the municipality of Presidente Prudente, mesoregion 8 [11] (Figure 1).

### 2.2. Demographic, Clinical, and Laboratory Characteristics of the Participants

This retrospective, long-term study cohort included 20 patients between January 2014 and December 2023 living in different municipalities of RNHA11, with a diagnosis of secondary immunodeficiencies, who were treated with different regimens. The patients were followed in the immunodeficiency outpatient clinic of the Regional Hospital of Presidente Prudente and in a specialized private clinic located in Presidente Prudente, Sao Paulo state, Brazil. Participants’ demographics, baseline clinical characteristics, different treatment regimens, and laboratory data, including serum electrophoresis, immunophenotyping blood counts, and immunoglobulin (Ig) levels after chemotherapy, were abstracted from electronic health records. Infections were further classified as organ-specific infections and severity was graded as mild, moderate, life-threatening, or fatal. Mild infection was defined as requiring oral antimicrobial therapy; moderate infection included the patient being hospitalized; life-threatening infection was defined as the presence of end-organ or cardiovascular compromise and treatment in Intensive Care Unit; and, in fatal infection, the patient died.

### 2.3. Immunoglobulin Replacement

Immunoglobulin replacement, endovenous or subcutaneous, when indicated, was given at a 28-day interval, and few patients received IgG replacement at intervals longer than 28 days. Patients followed at RH received different brands and quantities of immunoglobulin (5 g/50 mL or 5 g/100 mL) as provided by SUS. Patients treated in the private clinic received Endobulin Kiovig 10% (Imported by Takeda Pharma Ltda. Jaguariúna -SP, Brazil/ Produced by Baxalta Belgium Manufacturing S.A. Lessines, Belgium) or recombinant human hyaluronidase-facilitated subcutaneous immunoglobulin HyQvia (Imported by Takeda Pharma Ltda. Jaguariúna -SP, Brazil/Produced by Baxalta Belgium Manufacturing S.A. Lessines, Belgium). The initial dose was 400 mg/kg and was adjusted when necessary.

### 2.4. Inclusion and Exclusion Criteria

Only patients defined with SAD were screened. There is an enigmatic border between primary and secondary immunodeficiencies. In these situations, the case was discussed with a professional from the Immunodeficiencies Reference Center of Children’s Hospital (ICr), HCFMUSP, São Paulo, Brazil. Patients treated in Presidente Prudente but followed in other centers were excluded.

### 2.5. Statistical Analysis

The results are shown as means ± standard deviation (SD) (for normally distributed variables) and confidence intervals (95% CIs). Dichotomous and nominal variables are expressed as frequencies and percentages. For the immunologic markers, the different groups of patients were compared using a Kruskal–Wallis non-parametric test. A multiple regression analysis was applied to verify the correlation between the immunologic markers IgG, IgA, CD-19, CD3^+^, CD4^+^, and CD8^+^ and the number of infections in each patient studied. Statistical analysis was performed using GraphPad (San Diego, CA, USA, version 8.2.1) and GraphPad InStat 3, version 3.0a (San Diego, CA, USA).

### 2.6. Study Approval

This study was approved by the Ethics Committee of the University of Oeste Paulista, Presidente Prudente, São Paulo, Brazil (number 8008; 8 November 2023).

## 3. Results

### 3.1. Demographics, Clinical, and Laboratory Characteristics of the Participants at Baseline

The most frequent form of SAD was NHL (30%), followed by NS (25%), CLL (20%), MM (15%), protein-losing enteropathy (5%), and ITP (5%). The mean age was 41.87 ± 27.63 years (95% CI, 28.56–55.19 years). When distributed by groups, patients with NS were younger than patients with NHL (13.40 ± 4.82 years; interquartile range [IQR], 7.40–19.39 years vs. 66.83 ± 11.02 years; IQR, 55.26–78.39 years), CLL (66.75 ± 11.35 years; IQR, 47.68–83.82 years), and MM (52.0 ± 10.58; IQR, 25.71–78.29 years). Males were more prevalent than females with a ratio of 1.5:1, and European descendants were found in higher numbers. Hematologic malignancies accounted for 13 patients (65.0%), followed by autoimmune diseases in 6 patients (30.0%) and metabolic disorders in 1 patient (5.0%). At baseline, the mean IgG levels were 389.6 ± 185.20 mg/dL (95% CI, 302.9–476.3 mg/dL), and 60.0% of patients had an IgG level <400 mg/dL. The mean IgA level was 52.04 ± 54.46 mg/dL (95% CI, 24.04–80.05 mg/dL), and 55.0% of the patients had an IgA level < 40 mg/dL. Patient 6 was excluded due to MM hyper-γ-globulinemia induced by IgA (2.210 mg/dL). Of 17 patients available for γ-globulins, 16 (94.1%) had hypo-γ-globulinemia. The mean γ-globulin levels were 0.357 ± 0.20 (95% CI, 0.25–0.46). The mean CD19 level was 120.0 ± 156.2 cells/mm^3^ (95% CI, 39.71–200.3 mm^3^) (normal range in children, >200 mm^3^; adults, >140 mm^3^) (Table 1). The mean number of CD3^+^ T lymphocytes was 1743 ± 1362 cells/mm^3^ (IQR, 1017–2468 cells/mm^3^). The mean number of CD4^+^ T lymphocytes was 660.9 ± 544.1 cells/mm^3^ (IQR, 371.0–950.9 cells/mm^3^) and the mean number of CD8^+^ T lymphocytes was 999.9 ± 958.2 cells/mm^3^ (IQR, 489.3–1520 cells/mm^3^). When we compared the levels of IgG, IgA, γ-globulins, and CD19 between different groups of patients using the Kruskal–Wallis non-parametric test, the *p* value was non-significant for all the groups (0.372, 0.315, 0.228, and 0.172, respectively).

Multiple regression analysis was applied to verify the correlation between the immunologic markers IgG, IgA, CD-19, CD3^+^, CD4^+^, and CD8^+^ and the number of infections in each patient studied. The result was insignificant, presenting a correlation coefficient *r* = 44.6% and *p* > 0.68; the covariates also presented *p* values >0.05. A possible explanation for these results is the low number of patients and discrepancies in the values for the immunologic markers: normal IgG, IgA, and CD19 levels for patients with a high number of infections and decreased levels of IgG, IgA and CD19 for patients with fewer infections.

**Figure 2:**
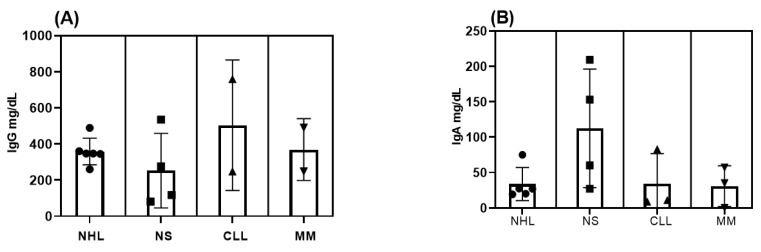

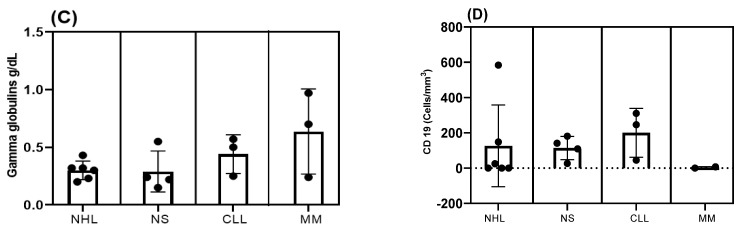
Immunologic markers in different groups of patients with secondary antibody deficiency after treatment with immunosuppressors and before replacement with immunoglobulins. NHL, non-Hodgkin’s lymphoma; NS, nephrotic syndrome; CLL, chronic lymphocytic leukemia; MM, multiple myeloma. IgG (**A**); IgA (**B**); γ-globulins (**C**); and CD-19 (**D**) levels.

There were no differences between public and private services regarding the origin of the patients and the replacement of immunoglobulin. The prevalent route of replacement of immunoglobulin was endovenous; however, four patients received the subcutaneous form (Table 1). Concerning treatment, RTX was used in 9 (45%) patients (Table 2). Immunological markers were analyzed separately (Figure 2). Most patients showed IgG levels < 400 mg/dL in all groups, but notably in the NHL group (Figure 2A). Similar results were found for IgA and γ-globulin levels (Figure 2B,C). In the patients with hematologic malignancies, after treatment with RTX/ibrutinib and teclistamab, 91.0% showed hypo-γ-globulinemia, 84.7% had low levels of IgG, and 72.3% had low levels of CD19. However, when CD19 was analyzed, these cells were not found in 4/6 (67.0%) patients with NHL (Figure 2D). Considering the main risk factors for NHL (age, >65 years old; gender, male, ethnicity, white), 50.0% of our patients were older than 65 years, all of them were white, and 50.0% were men.

### 3.2. Impact of Infections on Patients with Secondary Antibody Deficiency after Treatment with Immunosuppressants

**Figure 3:**
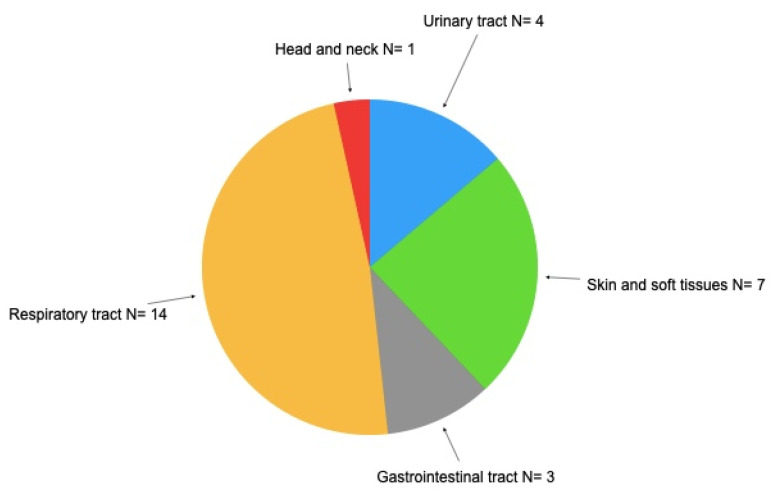
Distribution of infections by organs.

Table 3 shows the impact of infections at baseline after treatment with immunosuppressants and before the replacement of intravenous immunoglobulin. In 20 patients analyzed, there were 101 infection events (71 bacterial, 23 viral, and 7 fungal). Most of the infections, 83/101 (82.1%) were classified as mild or moderate, 8 (7.92%) as life-threatening, and 3 (3.0%) as fatal. Pneumonia was the most prevalent infection and accounted for 21 cases (20.7%); 9 cases of herpes simplex (8.9%) and 7 cases of herpes zoster, and 7 cases of skin mycosis (6.9%). When the infections were distributed by organ involvement, respiratory tract infections were more prevalent, accounting for 37 cases (36.6%), followed by 21 cases of skin disease and 21 cases of soft tissue infection (20.8%). A significant number of infections with dengue virus (2.9%) and COVID-19 (6.9%) was found. The number of patients with infections by organs is shown in Figure 3. A higher mean number of infections distributed by the diagnosis group was found for NHL, 7.00 ± 3.89 (IQR, 2.90–11.09), followed by 5.25 ± 4.64 (IQR, −2.13 to 12.64) for CLL, 3.66 ± 3.51 (IQR, −5.05 to 12.39) for MM, and 3.00 ± 4.47 (IQR, −2.55 to 8.55) for NS. Patients with NS showed significantly lower levels of infections than those with NHL (43 vs. 15; *p* = 0.0001). Patient 8 is a child who moved from Presidente Prudente to another state and was lost to follow-up, patient 16 was also lost to follow-up. Patient 19 died at the age of 6 months.

### 3.3. Infections in Different Sites in the Context of Gender, Age, and SAD Type

When the number of infections in different sites in the patients diagnosed with SAD in the context of the patient’s gender was analyzed, infections were found to be more prevalent in females (n = 8; 49 events) in a ratio of 6.1:1 than in males (n = 12; 47 events) in a ratio of 3.9:1; with a mean of 6.12 ± 4.91; (IQR, 2.01–10.23) for females and 4.27 ± 4.2 (IQR, 1.42–7.12) for males. Regarding age, patients between 18 and 60 years showed higher levels of infections (n = 7; 56 events) in a ratio of 8:1 compared with patients <18 years (n = 7; 30 events), and ≥60 years (n = 6; 26 events), both in a ratio of 4.3:1, with a mean of 8.00 ± 5.85 (IQR, 2.58 to 13.42) for patients aged between 18 and 60 years versus 4.28 ± 5.55 (IQR, 0.85–9.42) for those <18 years and 3.85 ± 2.73 (IQR, 1.32–6.38) for patients ≥ 60 years. Analyzing the infections presented by the patients in the light of immunologic markers, we found that patients with IgG levels < 400 mg/dL showed increased levels of infection (6.08 ± 4.81; IQR, 3.02–9.14) compared with those with IgG >400 mg/dL (3.28 ± 3.54; IQR, 0.006–6.56). In the same way, patients with IgA levels <40 mg/dL showed higher levels of infections (7.00 ± 5.04; IQR, 2.78–11.22) than patients with IgA levels > 41 mg/dL (2.14 ± 2.61; IQR, 0.27–4.55). Examining the role of CD19 in the context of infectious diseases, we found no differences between patients with CD19 levels lower and higher than 200 cells/mm^3^, with a mean of 5.00 ± 4.51 (IQR, 2.13–7.86) and 5.75 ± 4.78 (IQR, −1.86 to 13.37), respectively.

Table 4 with basal levels of the humoral immune response, obtained for each patient, before treatment with immunosupressants was constructed and inserted as a supplementary material. 

## 4. Discussion

The main findings of the study show that onco-hematologic malignancies and autoimmune diseases accounted for 65% and 30% of cases, respectively; hypo-γ-globulinemia was present in 94.1%, and 101 infection events were found.

Regarding the demographic characteristics of the study population, the patients with NS were younger than those with NHL, CLL, and MM. NS is the most common glomerular disease in the pediatric age group, and most patients progress until adulthood [12]. Age is the single biggest risk factor for hematologic malignancies. In Europe, the median age at diagnosis across all hematologic malignancies is 69 years and the incidence generally increases with age, reaching a maximum at 75–99 years [13]. In our study, males were found to be more prevalent than females in a ratio of 1.5:1. Males are at an increased risk of hematologic malignancies and have a worse prognosis with consistently poorer survival compared with females. According to the GLOBOCAN statistics, currently, 304,151 males versus 240,201 females have NHL; 98.613 versus 77.791 have MM [14]. In a study conducted in Martinica, the incidence of hematologic malignancies was higher in males, with rates close to 6 per 100,000 for MM and NHL, compared with 5 per 100,000 for females [15]. In São Paulo state, the adjusted rate of incidence of NHL per 100,000 men is estimated to be 5.38–9.47 for males and 3.60–359 for females for 2023 [16]. The reasons for the increased susceptibility for hematologic malignancies in men are not well-understood. In a recent review, possible causes include differences in environmental exposures, lifestyle, endogenous hormones, sex chromosomes, epigenetics, and probably complex multidirectional interactions between these factors [17]. In our cohort, the underlying risk factors were not addressed. Another interesting finding was that 50% of the patients were European and 10% were of Asian descent. The ethnic and racial composition of Brazilian society is the result of a confluence of people from several different ethnic origins, making it difficult to compare with other countries. No differences were found in the number of patients treated in the private specialized clinic compared with the public reference center, highlighting the role of RH in the diagnosis and treatment of patients with SAD in the context of RRAS11.

Hematologic malignancies accounted for 65.0% of cases of SAD; NHL was more prevalent (30.0%) than CLL (20%) and MM (15%). In Brazil, NHL is also the more prevalent hematologic malignancy. In 2023, 10,180 new cases of NHL were diagnosed in the country and were more prevalent in men [14]. According to GLOBOCAN, in 2020, the incidence of NHL was 544,352 cases and 176,404 cases for MM [14]. However, these results are different from studies on hematologic malignancies in Martinique, in which 47.8% were MM and 36% were NHL [15]. Due to a diverse class of B-cell and T-cell proliferation, NHL is the most common hematologic malignancy worldwide, accounting for nearly 3% of cancer diagnoses and deaths; it is the seventh most prevalent cancer and has the sixth highest mortality rate among cancers in the United States [17]. Among the non-modifiable risk factors, age >65 years has a more than double cumulative lifetime risk; white and non-Hispanic race/ethnicity has a higher risk; and family history, immunosuppression, and autoimmune diseases have been associated with various subtypes of NHL [17]. All our patients with NHL were white, representing the only risk factor, and only one patient was diagnosed with autoimmune disease (hypothyroidism). No previous immunosuppression was found before the diagnosis of NHL and treatment with RTX.

In our patients with hematologic malignancies, after treatment with RTX/ibrutinib and teclistamab, 91.0% showed hypo-γ-globulinemia, 84.7% had low levels of IgG, and 72.3% had low levels of CD19. Our findings on hypo-γ-globulinemia are higher than reported in other studies in which persistent or transient hypo-γ-globulinemia was expected after treatment with anti-CD20 biologics. In a study on RTX-associated hypo-γ-globulinemia in patients with multi-system autoimmune disease, 56% had hypo-γ-globulinemia during follow-up [18]. In a large cohort of patients treated with RTX who presented normal, mild, or moderate hypo-γ-globulinemia before treatment, 63.9% evolved to moderate or severe hypo-γ-globulinemia after RTX treatment [19,20]. One reason why these results were so different from ours may be the different pathologies among the patients. Different autoimmune diseases were screened, and the subgroups included hematologic malignancies (NHL and CLL), autoimmune/rheumatologic diseases, hematologic conditions, and primary immunodeficiencies [18,21]. Some probable mechanisms are suggested for RTX-induced hypo-γ-globulinemia, such as the impaired immune recovery that halted the differentiation from naive to memory B cells, increased B-cell apoptosis, and altered T-lymphocyte homeostasis [18]. When we submitted the different immunologic markers to the statistical analysis between groups, they were non-significant. When the immunologic markers IgG, IgA, CD19, CD3^+^, CD4^+^, and CD8^+^ were correlated with the number of individual infections, no correlation was found, either for each immunologic marker or considering all the parameters. Several factors contributed to these results, such as the great variation in values in each group, as well as the low number of patients in each group, resulting in low statistical power.

A total of 101 infections were registered in patients with SAD; 82.1% were classified as mild or moderate, 7.9% as life-threatening, and 3.0% as fatal (two patients died from sepsis; one patient died after COVID-19 infection). Our study presents similar results to those for patients treated with CART-cells therapy, in which 71% of all infections were considered mild to moderate [21]. In a large cohort of 1261 patients treated with RTX for different pathologies, 28.2% had severe infections after treatment [19]. In our study, pneumonia was the most prevalent infection followed by sinusitis. When the infections were distributed according to organ involvement, respiratory tract infections were more prevalent, followed by skin and soft tissue infections, triggered mainly by herpes simplex and herpes zoster viruses. Among hematologic malignancies, NHL showed a higher mean prevalence of infection, followed by CLL and MM. In line with our results, increased rates of respiratory tract infections, pneumonia, and sinusitis were demonstrated in patients with NHL treated with RTX in São Paulo state [6]. Respiratory tract infections were the most common infection observed in patients with SID treated with different biologics and immunomodulatory therapies, including tumor necrosis factor-α inhibitors, interleukin-12/interleukin-23 inhibitors, anti-T-lymphocyte therapies, and anti-B-lymphocyte therapies [19]. The mechanisms underlying RTX-induced respiratory tract infections are unclear, but abnormalities in IL-2 production and IL-2R expression, associated with decreased antigen-induced lymphocyte proliferation, late-onset neutropenia, and delayed onset cytopenia, are described [8].

One of the most important findings in our study was the significant number of patients with NS accounting for 30.0% of cases of infection. Four of our patients (80.0%) were treated with RTX; however, hypo-γ-globulinemia, low levels of IgG, and CD19 were present in all patients before the treatment. Although hypo-γ-globulinemia in NS is regarded as a risk factor for infections, our patients showed lower levels compared with NHL (*p* = 0.0001). Our results are in line with 140 patients with childhood-onset idiopathic steroid-sensitive nephrotic syndrome treated with RTX; hypo-γ-globulinemia was not associated with an increase or the severity of infections [12]. NS is characterized by proteinuria, hypoalbuminemia, hyperlipidemia, and generalized edema, and hypo-γ-globulinemia is a frequent finding. Some probable mechanisms leading to this phenomenon are suggested, such as the relapsing course, often triggered by infections or allergies, the response to immunosuppressive regimens, such as steroids, cyclophosphamide, and cyclosporine, and the absence of structural abnormalities of the glomerular basement membrane. Hypo-γ-globulinemia was believed to be caused by the urinary loss of IgG; however, it is known that the parallel increase in IgM suggests a defect in the switch from IgM to IgG synthesis due to an unknown immunologic defect, leading to low levels of immunoglobulins [12].

In contrast to the scientific literature on immunocompetent individuals, in the context of the patient’s gender, we found that infections were more prevalent in women than in men. For immunocompetent individuals, the importance of gender in their susceptibility to infectious diseases is well-known. Women typically develop higher innate, humoral, and cellular immune responses to viral infections, but are more susceptible to autoimmune diseases. Men are generally more susceptible than women to bacterial infections. Genetic, immunologic, hormonal, and anatomic factors may be related to and influence sex-based differences in the severity and prognosis of infectious diseases [22]. However, few data are available that assess the vulnerability to infections in people with primary or secondary immunodeficiency based on gender. Regarding age, patients between 18 and 60 years of age had higher levels of infections than younger (<18 years) and older (≥60 years) patients. In clinical practice, the severity of infections in young or middle-aged people is anecdotal. Young people have a great immune response to most infectious diseases and rarely get sick. When this happens, all defense barriers have probably been broken, and the patient tends to progress quickly and severely. Clinicians should be aware of young and middle-aged people with viral or bacterial pneumonia, glomerulonephritis, influenza, dengue fever, or COVID-19 infections [23]. Regarding immunologic markers, we found that patients with IgG levels <400 mg/dL had increased levels of infection compared with those with IgG >400 mg/dL, as well as patients with lower IgA levels. In line with our results, it is well-known that, in EII and SID, patients with low IgG levels alone or associated with low IgA or IgM levels are susceptible to respiratory tract infections and recurrent sinusitis [19].

The main strength of this study was the inclusion of different types of SAD in the cohort who received different immunosuppressive agents. As far as we know, no data with these characteristics have been published before in Brazil. Our region harbors a great number of small municipalities in a developing region with a regional health reference center; therefore, the study has global relevance and may be applied in Brazilian regions or countries with similar social and epidemiological characteristics to RRAS11. 

Several shortcomings must be mentioned. Some immunologic data were lost because some patients moved from RRAS-11 or died at the beginning or during the follow-up. For some patients, there is a lack of basal results on cellular and humoral immune responses before treatment with immunosuppressors. The duration of the study varied for each patient, generating a bias in the results. Some groups had very few patients such as MM, autoimmune thrombocytopenia, and protein-losing enteropathy. A functional immune study of vaccine response, which is the basis for assessing primary or secondary immunodeficiencies beyond the decrease in immunoglobulins, is lacking. The low number of patients assessed in these analyses allows for low statistical power. It is a retrospective analysis, subject to selection and misclassification bias.

## 5. Conclusions

In our cohort, most patients progressed to hypo-γ-globulinemia and low levels of IgG, IgA, and CD19 after treatment with immunosuppressants. Respiratory tract infections were the more prevalent and pneumonia was the main infection; mild and moderate infections were predominant. In the context of the patient’s gender and age, infections were found to be more prevalent in females and in young and middle-aged patients. Regarding immunological markers, patients with low levels of IgG and IgA showed higher levels of infections, highlighting the role of immunosuppressants in inducing hypo-γ-globulinemia. These therapies are increasing in different diseases, and in developing countries; therefore, monitoring hypo-γ-globulinemia and infections may help to identify patients at high risk for severe complications by introducing antibiotic prophylaxis or treatment, and immunoglobulin replacement. Our study opens up perspectives for future publications. From this database, new patients are being incorporated into both public and private services, and new immunologic markers are being determined. Furthermore, the involvement of different professionals from the western region of São Paulo has increased the number of patients investigated for SID, and the region can become a reference center for these pathologies. Furthermore, the study shows the importance of public reference hospitals in the diagnosis and treatment of SID patients from SUS in Brazil.

## Figures and Tables

**Table 1:** Immunologic markers, pathology, cell target therapy, and drug used.

No.	IgG	IgA	γ-Globulins	CD19	CD3^+^	CD4^+^	CD8^+^	Path	Target	Drug
1	360	27	0.30	0	590	273	311	NHL	Anti-CD-20	RTX
2	489	75	0.43	584	1129	467	577	NHL	Anti-CD-20	RTX
3	260	27.2	0.23	25	1877	482	1337	NHL	Anti-CD-20	RTX
4	345	19.2	0.32	0	5025	1044	3704	NHL	Anti-CD-20	RTX
5	347	20	0.32	148	878	371	392	NHL	BTK	Ibrut
6	347	2210	0.20	00	1297	324	877	NHL	BTK	Ibrut
7	117	153	0.22	141	406	251	100	NS	Anti-CD-20	Pred
8	320	NA	NA	NA	NA	NA	NA	NS	Anti-CD-20	Pred
9	535	209	0.55	26	1188	343	808	NS	IMPDH	Pred; myc
10	276	27	0.24	109	1102	650	421	NS	Anti-CD-20	Pred
11	81	60	0.15	181	4374	2011	2052	NS	Anti-CD-20	Pred
12	248	10.9	0.24	246	2923	435	2408	CLL	Anti-CD-20	RTX
13	654	8.73	0.70	45	855	334	470	CLL	Anti-CD-20	RTX
14	759	83	0.97	310	NA	NA	NA	CLL	Anti-CD-20	RTX
15	415	NA	NA	NA	NA	NA	NA	CLL	Anti-CD-20	RTX; ibrut
16	490	35	0.50	NA	NA	NA	NA	MM	DNA	Cycloph; tali
17	248	57	0.57	7	643	278	329	MM	Anti-CD-20	RTX
18	282	0	0.25	0	863	345	499	MM	BCMA/CD3	Teclistamab
19	76	33	0.11	218	2309	1600	772	PD	0	No drugs
20	460	39.7	0.35	0	2425	1367	941	ITP	Anti-CD-20	RTX

IgG (mg/dL); IgA (mg/dL); γ-globulins (g/dL); CD19 (cells/mm^3^); CD3^+^ (cells/mm^3^); CD4^+^ (cells/mm^3^); CD8^+^ (cells/mm^3^). Path, pathology; NHL, non-Hodgkin’s lymphoma; BTK, Bruton tyrosine kinase; NS, nephrotic syndrome; IMPDH, inosine monophosphate dehydrogenase; CLL, chronic lymphocytic leukemia; MM, multiple myeloma; BCMA, B-cell maturation antigen; PLE, protein-losing enterophaty; ITP, immune thrombocytopenia; NA, not available. RTX, rituximab; Ibrut, ibrutinib; Pred, prednisone; Cycloph, cyclophosphamide; Tali, Talidomid.

**Table 2:** Patient demographics, and clinical characteristics.

	Participants (n= 20)	n (%)	Mean ± SD	95% Confidence Interval
Age (years)	<18	7 (35)	10.80 ± 7.29	4.05–17.54
	18–60	7 (35)	49.29 ± 12.74	37.51–61.07
	≥60	6 (30)	70.83 ± 8.25	62.17–79.50
Gender	Female	8 (40)		
	Male	12 (60)		
Race	European	10 (50)		
	South American	6 (30)		
	African	2 (10)		
	Asian	2 (10)		
SID type	Non-Hodgkin’s lymphoma	6 (30)		
	Nephrotic syndrome	5 (25)		
	Chronic lymphocyte leukemia	4 (25)		
	Multiple myeloma	3 (15)		
	Protein-losing enteropathy	1 (5)		
	Immune thrombocytopenia	1 (5)		
Origin of the service	Public	10 (50)		
	Private	10 (50)		
IgG replacement	Endovenous	12 (60)		
	Subcutaneous	4 (25)		
	Not indicated	4 (25)		

SD, standard deviation; SID, secondary immunodeficiency.

**Table 3:** Infections in different sites in patients diagnosed with secondary antibody deficiency (2014–2023).

	Patient Number																			
	1	2	3	4	5	6	7	8	9	10	11	12	13	14	15	16	17	18	19	20
Infection sites																				
Upper and lower airway infections and otitis																				
Tonsilitis	**^_^**	**^_^**	**^_^**	**^_^**	**+**	**+++**	**^_^**	**^_^**	**^_^**	**^_^**	**^_^**	**^_^**	**^_^**	**++**	**++**	**++**	**^_^**	**+**	**^_^**	**+**
Sinusitis	**++++**	**^_^**	**^_^**	**^_^**	**^_^**	**^_^**	**^_^**	**^_^**	**^_^**	**^_^**	**^_^**	**^_^**	**^_^**	**^_^**	**^_^**	**+**	**^_^**	**^_^**	**^_^**	**^_^**
Otitis			**^_^**	**^_^**	**^_^**	**^_^**	**^_^**	**^_^**	**^_^**	**^_^**	**^_^**	**+**	**^_^**	**^_^**	**^_^**	**^_^**	**^_^**	**^_^**	**^_^**	**^_^**
Pneumonia	**+++**	**++**	**^_^**	**++++**	**^_^**	**^_^**	**^_^**	**^_^**	**^_^**	**++++**	**^_^**	**^_^**	**^_^**	**^_^**	**^_^**	**^_^**	**^_^**	**+++**	**+++**	**^_^**
Skin diseases and soft tissues																				
Herpes simplex	**++**	**^_^**	**^_^**	**^_^**	**+++**	**^_^**	**^_^**	**^_^**	**^_^**	**^_^**	**^_^**	**+++**	**+**	**^_^**	**^_^**	**^_^**	**^_^**	**^_^**	**^_^**	**^_^**
Herpes zoster	**^_^**	**^_^**	**^_^**	**^_^**	**^_^**	**^_^**	**^_^**	**^_^**	**^_^**	**++**	**^_^**	**+**	**+**	**^_^**	**+++**	**^_^**	**^_^**	**^_^**	**^_^**	
Skin mycosis	**^_^**	**^_^**	**^_^**	**^_^**	**^_^**	**^_^**	**^_^**	**^_^**	**++++**	**^_^**	**^_^**	**^_^**	**^_^**	**^_^**	**+++**	**^_^**	**^_^**	**^_^**	**^_^**	
Head and neck																				
Tongue lesion	**^_^**	**^_^**	**^_^**	**^_^**	**^_^**	**^_^**	**^_^**	**^_^**	**^_^**	**^_^**	**^_^**	**++**	**^_^**	**^_^**	**^_^**	**^_^**	**^_^**	**^_^**	**^_^**	**^_^**
Gastrointestinal																				
Gastroenteritis	**+**	**^_^**	**^_^**	**^_^**	**^_^**	**^_^**	**^_^**	**^_^**	**^_^**	**^_^**	**^_^**	**+**	**^_^**	**^_^**	**+++**	**^_^**	**^_^**	**^_^**	**++++**	**+**
Urinary tract																				
UTI	**+++**	**++**	**^_^**	**^_^**	**^_^**	**^_^**	**^_^**	**^_^**	**^_^**	**++++**	**^_^**	**^_^**	**^_^**	**^_^**	**^_^**	**^_^**	**^_^**	**^_^**	**++**	**^_^**
Sepsis																				
Sepsis	**+**	**^_^**	**^_^**	**+**	**^_^**	**^_^**	**^_^**	**^_^**	**+**	**++**	**^_^**	**^_^**	**^_^**	**^_^**	**^_^**	**^_^**	**^_^**	**++**	**+++**	**^_^**
Dengue and COVID-19																				
Dengue			**+**		**+**	**^_^**	**^_^**	**^_^**	**^_^**	**^_^**	**^_^**	**^_^**	**^_^**	**^_^**	**^_^**	**^_^**	**^_^**	**^_^**	**^_^**	**^_^**
COVID-19	**+**	**+**	**++**	**+**	**+**	**++**	**^_^**	**^_^**	**^_^**	**^_^**	**^_^**	**^_^**	**^_^**	**^_^**	**^_^**	**^_^**	**^_^**	**^_^**	**^_^**	**^_^**
Severity of infections																				
Mild	**^_^**	**^_^**	**+**		**+**	**^_^**	**^_^**	**^_^**	**^_^**	**^_^**	**^_^**	**+**	**+**	**+**		**+**				**+**
Moderate	**^_^**	**+**	**^_^**	**^_^**	**^_^**	**^_^**	**^_^**	**^_^**	**^_^**	**^_^**	**^_^**	**^_^**	**^_^**	**^_^**	**^_^**	**^_^**	**^_^**	**^_^**	**^_^**	**^_^**
Life-threatening	**+**	**^_^**	**^_^**	**+**	**^_^**	**+**	**^_^**	**^_^**	**+**	**+**	**^_^**	**^_^**	**^_^**	**^_^**	**+**	**^_^**	**^_^**	**+**	**+**	**^_^**
Fatal	**+**	**^_^**	-	**^_^**	**^_^**	**^_^**	**^_^**	**^_^**	**^_^**	**^_^**	**^_^**	**^_^**	**^_^**	**^_^**	**+**	**^_^**	**^_^**	**^_^**	**+**	**^_^**

(**+**), (**++**), (**+++**), and (**++++**) indicate the number of infections related to each patient. (**^_^**) means that this type of infection has not been observed. UTI, urinary tract infection.

**Table 4:** Basal Levels of humoral immune response before treatment with immunosuppressants.

Patient No.	Basal IgG	IgG	Basal IgA	IgA	Basal γ-glo	γ-glob
1	753	360	41	27	NA	0.30
2	540	489	73	75	0.70	0.43
3	480	260	35	27.2	NA	0.23
4	360	345	23	19.2	0.50	0.32
5	447	347	27.1	20	NA	0.32
6	410	347	2430	2210	31.10	0.20
7	125	117	170	153	0.25	0.22
8	NA	320	NA	NA	NA	NA
9	586	535	223	209	0.81	0.55
10	235	276	59.6	27	0.30	0.24
11	65	81	71	60	0.25	0.15
12	950	248	<5.0	10.9	NA	0.24
13	906	654	11.9	8.73	NA	0.70
14	NA	759	NA	83	NA	0.97
15	NA	415	NA	NA	NA	NA
16	NA	490	NA	35	NA	0.50
17	516	248	2693	57	0.45	0.57
18	310	282	<5.0	0	0.48	0.25
19	217	76	35	33	0.23	0.11
20	490	460	45	39.7	0.70	0.35

NA, not available; IgG (mg/dL); IgA (mg/dL); γ-globulins (g/dL).

## Data Availability

All data may be shared and should be requested from the corresponding author.
